# From Single Agents to Synergy: Redefining Therapeutic Strategies in MASLD

**DOI:** 10.3390/ijms27188368

**Published:** 2026-09-20

**Authors:** Lampros Chrysavgis, Evangelos Cholongitas

**Affiliations:** First Department of Internal Medicine, General Hospital Laiko, Medical School, National and Kapodistrian University of Athens, AgiouThoma 17, 11527 Athens, Greece; lchrisaugis@gmail.com

**Keywords:** metabolic dysfunction-associated steatotic liver disease (MASLD), resmetirom, semaglutide, fibrosis, randomized clinical trials, combination treatment, precision medicine

## Abstract

The recent approvals of resmetirom and semaglutide have marked a pivotal advance in the management of metabolic dysfunction-associated steatotic liver disease (MASLD). Nevertheless, the modest fibrosis responses observed with current monotherapies and the persistence of substantial residual disease burden highlight the need for more comprehensive therapeutic strategies. Given the multifactorial pathogenesis of MASLD, combination therapy has emerged as a promising approach to simultaneously target metabolic dysfunction, hepatic steatosis, inflammation, and fibrogenesis. In this opinion article, we discuss the biological rationale for combination treatment and critically appraise the evidence from randomized clinical trials evaluating multidrug approaches in MASLD. While several studies have demonstrated encouraging improvements in hepatic and metabolic outcomes, others have failed to achieve their primary endpoints, underscoring that mechanistic complementarity does not necessarily translate into clinical synergy. We further review ongoing trials, including studies in advanced fibrosis and compensated cirrhosis, that are expected to shape the next phase of therapeutic development. Finally, we address key challenges related to patient selection, disease heterogeneity, treatment duration, safety, cost-effectiveness, and regulatory approval. We propose that the future of combination therapy in MASLD will likely rely on precision-medicine approaches that align therapeutic mechanisms with individual disease biology and stage.

## 1. Introduction

**Figure 1:**
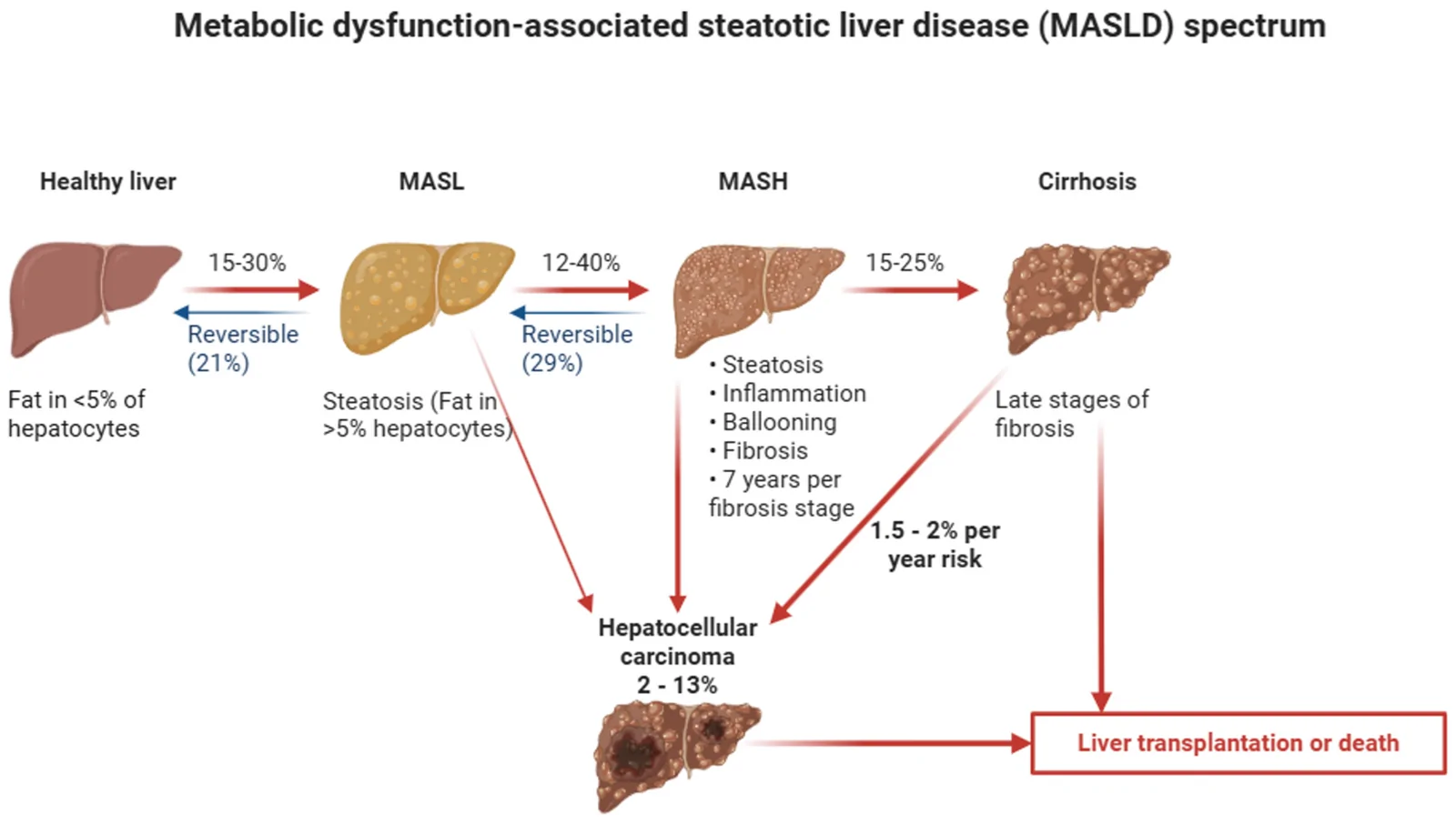
Natural history of metabolic dysfunction-associated steatotic liver disease (MASLD). Abbreviations: MASH, metabolic dysfunction-associated steatohepatitis; MASL, metabolic dysfunction-associated steatotic liver.

The global burden of liver disease has undergone a major shift. As viral hepatitis is increasingly controlled, metabolic dysfunction-associated steatotic liver disease (MASLD) has emerged as the most common chronic liver condition worldwide, reflecting the global epidemic of obesity, insulin resistance and type 2 diabetes mellitus (T2DM) [1]. The 2023 nomenclature change from non-alcoholic fatty liver disease (NAFLD) to MASLD [2] was not solely a matter of semantics; it formalized the medical community’s recognition of the disease as a manifestation of systemic metabolic dysfunction [3]. The prevalence of MASLD has risen by more than 50% over the past three decades, now affecting approximately 38.2% of adults worldwide and representing by far the most common form of steatotic liver disease [4,5]. While MASLD is considered a benign disease, a subset of patients progresses to a more virulent histological phenotype, namely metabolic dysfunction-associated steatohepatitis (MASH), characterized by hepatocellular injury, inflammation, and progressive fibrosis [6]. The latter may subsequently progress to advanced fibrosis and cirrhosis, substantially increasing the risk of hepatocellular carcinoma (HCC). Importantly, HCC may also arise in a minority of MASH patients in the absence of cirrhosis, reflecting the direct oncogenic effects of the underlying metabolic and inflammatory milieu [7,8] (Figure 1). Recent epidemiological projections are sobering. MASLD is the leading cause of liver transplantation (LT) in women in the United States and the second-most-common indication overall, underscoring its clinical urgency [9]. Concurrently, by 2030, the cases of decompensated cirrhosis and HCC are expected to rise by more than 50% globally, mainly attributed to MASLD/MASH [10].

The pathogenesis of MASLD involves interdependent processes: increased free fatty acid flux to the liver, adipose tissue dysfunction, hepatic de novo lipogenesis, mitochondrial dysfunction and oxidative stress, innate immune activation, and hepatic stellate cell-mediated fibrogenesis [11,12,13]. Additionally, systemic metabolic dysregulation—insulin resistance, dyslipidaemia, and chronic low-grade inflammation—amplifies hepatic injury [14]. Targeting a single node within this network may inadequately address the multifactorial drivers of the disease, particularly in patients with more advanced histology. Despite the vast scientific effort globally, effective pharmacotherapy has historically been absent. For decades, lifestyle modification—encompassing calorie restriction, with stricter adherence to the Mediterranean diet, and structured physical activity—remained the only cornerstone of management for MASH [15,16,17]. A reduction in body weight by 7–10% induces meaningful histological improvement, including MASH resolution in approximately 40–60% of cases [18]. However, long-term adherence is challenging, and sustained weight loss is attained by a minority of patients in real-world settings [19]. For patients with severe obesity and concurrent MASLD, metabolic and bariatric surgery represents an effective intervention that offers long-term disease modification by achieving profound, sustained weight loss. Long-term prospective data and meta-analyses confirm that metabolic surgery leads to the histological resolution of MASH in a significant proportion of patients [20] and drives significant regression of advanced liver fibrosis, reduces the risk of burdened hepatic and extrahepatic outcomes [21], as well as the risk of HCC [22]. Pharmacotherapy for MASH relied on older agents based on foundational trials like the PIVENS study, which demonstrated that vitamin E and pioglitazone could significantly improve steatosis and inflammation compared to placebo [23]. However, these treatments are now rarely utilized in modern clinical practice. Moreover, statins, as first-line treatment for atherogenic dyslipidemia, are primary tools for mitigating cardiovascular disease in patients with MASLD. Beyond that, clinical evidence underscores their substantial, direct hepatoprotective benefits since they exert anti-inflammatory, anti-fibrotic, and antioxidant effects [24,25] that decrease the severity of hepatic steatosis and slow the progression of liver stiffness. Furthermore, recent large-scale studies and meta-analyses confirm that sustained statin therapy is associated with a dose-dependent reduction in the incidence of MASLD-related HCC [26,27].

Concurrently, there have been several phase 2 and phase 3 randomized clinical trials (RCTs) characterized by the failure to demonstrate histologically significant reduction in inflammation and/or regression of fibrosis. The pharmacological landscape of MASLD is currently anchored by two distinct classes of drugs that have successfully met the surrogate histological endpoints required for conditional approval. Resmetirom, a liver-directed, selective agonist of the thyroid hormone receptor-beta(THR-β) (MAESTRO-NASH trial) [28], was the first drug that achieved Food and Drug Administration (FDA) approval for the treatment of MASH with moderate to severe fibrosis. Later, in 2025, resmetirom was also approved by the European Medicines Agency (EMA). More recently, interim results from the phase 3 ESSENCE trial [29] demonstrated that weekly semaglutide, a long-acting glucagon-like peptide-1 (GLP-1) receptor agonist (GLP-1RA) at a dose of 2.4 mg, significantly improved histological outcomes in patients with MASH and stage F2–F3 fibrosis after 72 weeks, supporting its accelerated FDA approval in August 2025 and EMA approval in March 2026. Yet even with these approved therapies, monotherapy is insufficient for a significant proportion of patients. The MAESTRO-NASH trial of resmetirom [28] demonstrated MASH resolution in 25.9–29.9% of patients and a ≥1-stage fibrosis improvement in 24.2–25.9%, while the ESSENCE trial [29] of semaglutide achieved MASH resolution in 62.9% and fibrosis improvement in 36.8% of participants. These response rates leave substantial proportions of patients with residual hepatic pathology, particularly fibrosis—the most predictive histological parameter of all-cause and liver-related mortality.

**Figure 2:**
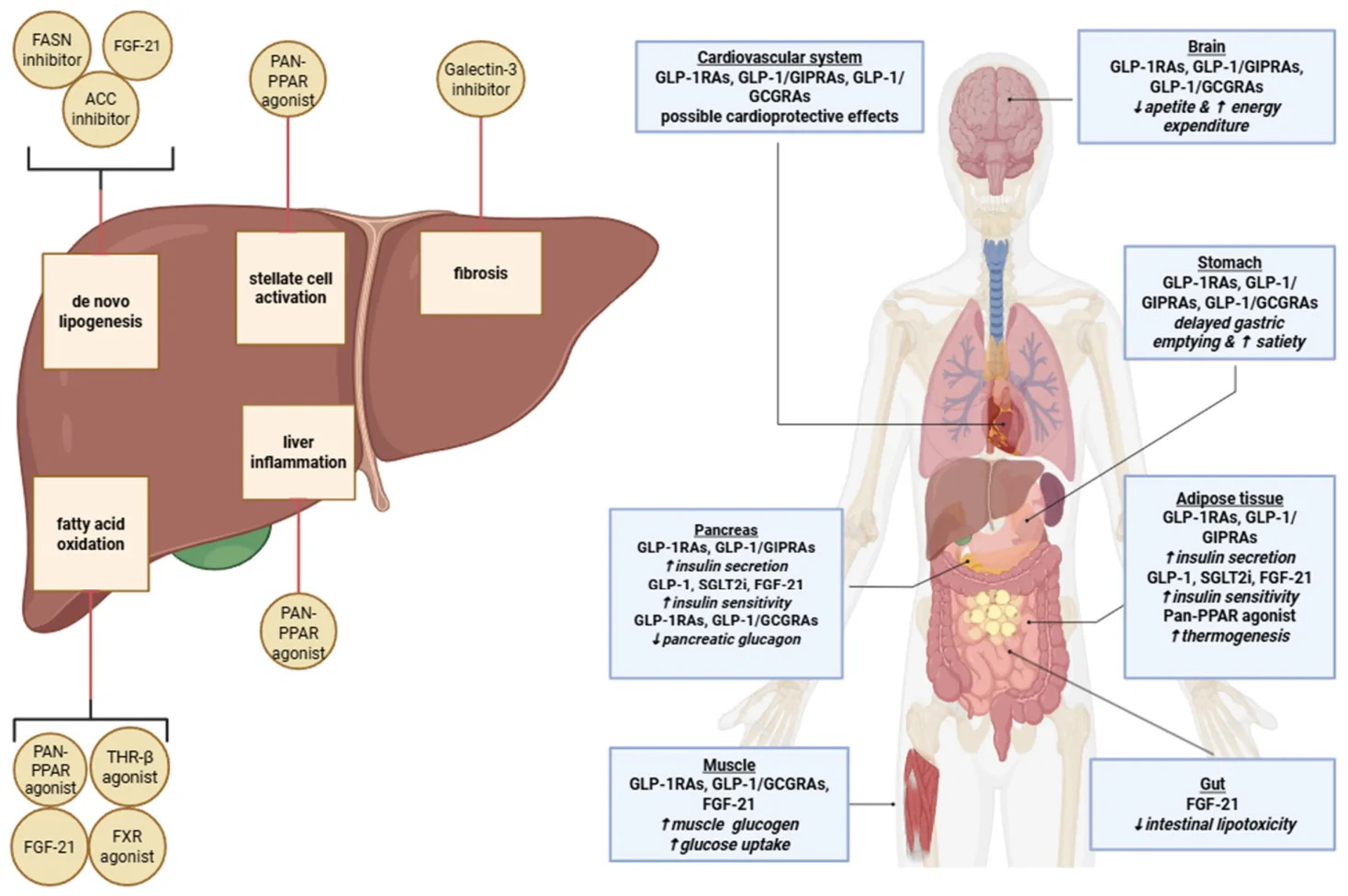
Mechanistic framework of metabolic-targeted and liver-targeted therapies in metabolic dysfunction-associated steatohepatitis (MASH). Abbreviations: ACC, acetyl-CoA carboxylase; FASN, fatty acid synthase; FGF-21, fibroblast growth factor 21; FXR, farnesoid X receptor; GCGR, glucagon receptor; GIP, glucose-dependent insulinotropic polypeptide; GIPRA, glucose-dependent insulinotropic polypeptide receptor agonist; GLP-1, glucagon-like peptide-1; GLP-1RA, glucagon-like peptide-1 receptor agonist; Pan-PPAR, pan-peroxisome proliferator-activated receptor; SGLT2i, sodium–glucose cotransporter-2 inhibitor; THR-β, thyroid hormone receptor beta; ↑, increased/greater; ↓, reduced/lower.

These observations highlight the multifactorial, multifaceted nature of MASLD and reflect the convergence of multiple pathogenic pathways that are not fully addressed by modulation of a single molecular target. The principal rationale for a potential combination therapy is to concurrently modulate distinct yet interconnected pathogenic pathways, thereby achieving complementary or synergistic effects that enhance overall therapeutic efficacy (Table 1 & Figure 2). Beyond potential gains in clinical response, systematic evaluation of combination regimens may enable more appropriate treatment selection, minimize unnecessary drug exposure, and mitigate cumulative toxicity and potential side effects.

This opinion article aims to critically appraise the evolving role of combination therapy in MASH, with particular emphasis on the biological rationale, the evidence generated by published RCTs, the increasing number of ongoing RCTs, and the challenges that must be addressed before these strategies can be integrated into routine clinical practice. To provide a comprehensive overview of the available evidence, a targeted literature search was conducted in the PubMed database, along with extracting data presented at major international hepatology congresses and scientific meetings. The search was conducted using combinations of the terms “MASLD”, “MASH”, “metabolic dysfunction-associated steatohepatitis”, “combination therapy”, “combination treatment”, “randomized controlled trial”, “clinical trial”, “RCT”, “GLP-1”, “dual incretin agonist”, “triple incretin agonist”, “FGF21”, “FXR”, “THR-β”, “PPAR”, “SGLT2”, “ACC inhibitor”, “DGAT2 inhibitor”, and “fibrosis”. The following sections summarize the current clinical evidence and discuss its implications for future therapeutic development.

## 2. Published Phase 2 and 3 RCTs for Combination Treatment in MASH

The published RCTs evaluating combination therapies in MASH are summarized in Table 2 and are discussed below according to their principal therapeutic strategy.

### 2.1. GLP-1/GIP Dual Agonist—Tirzepatide (SYNERGY-NASH)

Tirzepatide, a once-weekly dual GLP-1/GIP receptor agonist, was evaluated in the phase 2b SYNERGY-NASH trial in patients with biopsy-confirmed MASH and F2–F3 fibrosis. The polypeptide GIP acts synergistically with GLP-1 by enhancing glucose-dependent insulin secretion, thereby improving glycemic control and weight loss and regulating hepatic lipid metabolism [30]. The study demonstrated robust, dose-dependent improvements in MASH resolution together with encouraging effects on fibrosis, supporting the concept that simultaneous targeting of complementary incretin pathways may enhance histological response.

### 2.2. GLP-1/Glucagon (GCG) Dual Agonists

Dual GLP-1/GCG receptor agonists combine the metabolic benefits of GLP-1 receptor activation with glucagon-mediated enhancement of hepatic fatty acid oxidation and energy expenditure. Survodutide demonstrated significant improvements in both MASH resolution and fibrosis in a phase 2 RCT [31]. In the recently reported phase 3 SYNCHRONIZE-MASLD RCT, treatment with survodutide for 48 weeks resulted in significantly greater magnetic resonance imaging (MRI) proton density fat fraction (PDFF) assessed liver fat content reduction, compared to placebo, in 216 obese and at-risk (MRI-PDFF ≥ 8% at screening) MASLD patients [32]. Similarly, efinopegdutide in a phase 2a RCT produced substantially greater liver fat reduction than semaglutide despite comparable weight loss, suggesting direct hepatic benefits beyond body weight reduction [33]. In the IMPACT phase 2b, pemvidutide also achieved high rates of MASH resolution but failed to significantly improve fibrosis [34]. Building upon these findings, a late clinical-stage biopharmaceutical company will initiate a Phase 3 PERFORMA RCT that will evaluate pemvidutide in approximately 990 patients with biopsy-confirmed MASH and F2–F3 fibrosis, using histological endpoints at 52 weeks to support accelerated approval, while long-term follow-up (~60 months) will assess liver-related clinical outcomes. Notably, the study incorporates AI-assisted histological assessment (AIM-MASH AI Assist) to improve the standardization and reproducibility of liver biopsy interpretation.

### 2.3. GLP-1/GIP/GCG Triple Agonist—Retatrutide

Retatrutide, a triple GLP-1/GIP/GCG receptor agonist, achieved the largest pharmacologically induced reduction in liver fat reported to date in MASLD, with a clear dose–response relationship [35] in a phase 2a RCT. Although histological data are not yet available, these findings reinforce the remarkable metabolic efficacy of triple incretin agonism and support its ongoing phase 3 development (SYNERGY-Outcomes/NCT07165028).

### 2.4. GLP-1 Receptor Agonists Combined with THR-β Agonists

Resmetirom and semaglutide represent the first FDA-approved pharmacological therapies for MASH [28,29], providing a strong biological rationale for combination therapy. They are mechanistically complementary: resmetirom acts via hepatic THR-β to upregulate mitochondrial fatty acid oxidation through weight-independent intrahepatic mechanisms [36], whereas GLP-1RAs drive hepatic benefit primarily through systemic weight reduction and insulin sensitisation. A prespecified subgroup analysis of MAESTRO-NASH showed that resmetirom retained its efficacy irrespective of background GLP-1 receptor agonist use, although no additive benefit was observed [36]. Importantly, interpretation was limited by the use of earlier-generation GLP-1 receptor agonists, such as dulaglutide and liraglutide and relatively low semaglutide doses used for T2DM rather than obesity [36].

### 2.5. GLP-1 Receptor Agonists Combined with FGF21 Analogues

The addition of efruxifermin to stable GLP-1 receptor agonist (semaglutide, dulaglutide, or liraglutide) therapy produced marked reductions in liver fat and liver stiffness despite similar weight loss between treatment groups, suggesting additive liver-directed effects beyond metabolic improvement [37]. Consistent with these findings, a first-in-class GLP-1/FGF21 dual agonist demonstrated rapid dose-dependent reductions in hepatic steatosis, providing further proof-of-concept for this therapeutic approach [38]. Importantly, neither RCT incorporated paired liver biopsies or histological endpoints; consequently, the observed benefits were based exclusively on non-invasive imaging and biomarker assessments.

### 2.6. GLP-1 Receptor Agonists Combined with FXR Agonists and ACC Inhibitors

A proof-of-concept phase 2 study evaluated semaglutide in combination with cilofexor and/or firsocostat in 108 patients with MASH and F2–F3 fibrosis over 24 weeks. The combination treatment resulted in greater improvements in hepatic steatosis, liver biochemistry, and non-invasive fibrosis markers than semaglutide alone, despite similar weight loss across treatment groups [39], but not significant changes in liver fibrosis, as assessed by histology. These findings support complementary metabolic and liver-directed mechanisms and provided the rationale for subsequent evaluation in patients with compensated MASH cirrhosis.

### 2.7. Pan-PPAR Agonist Combined with Sodium–Glucose Cotransporter-2 Inhibitor (SGLT2) Inhibitor—LEGEND Trial

The phase 2 LEGEND trial evaluated lanifibranor with or without empagliflozin in patients with MASH and T2DM [40]. While both active treatment arms improved glycemic control and reduced liver fat, the combination uniquely prevented the weight gain typically associated with PPARγ activation while maintaining favourable hepatic and metabolic effects, illustrating how combination therapy may optimize both efficacy and tolerability. However, these promising proof-of-concept findings should be considered preliminary since they have only been presented at the “Liver Meeting” in 2024, and not yet been published in a peer-reviewed journal.

### 2.8. THR-β Agonist Combined with FXR Agonist—DUET Trial

The DUET trial investigated TERN-501, a THR-β agonist, alone or in combination with the FXR agonist TERN-101 [41]. Although the addition of TERN-101 did not further reduce liver fat, it improved fibro-inflammatory biomarkers. Importantly, a safety benefit was demonstrated: TERN-501 monotherapy raised LDL cholesterol as an on-target class effect, but the addition of TERN-101 fully attenuated this elevation, with the combination showing no net LDL increase from baseline. This safety-complementarity—FXR agonism mitigating the LDL-raising effect of THR-β agonism—represents a rational pharmacological pairing beyond efficacy alone. However, studies with longer duration and histological endpoints are required.

### 2.9. ACC Inhibitor Combined with DGAT2 Inhibitor—MIRNA Trial

The MIRNA trial evaluated the combination of ervogastat (DGAT2 inhibitor) and clesacostat (ACC inhibitor) in patients with MASH and F2–F3 fibrosis [42]. The combination achieved significantly higher rates of the composite histological endpoint, driven primarily by improvements in MASH resolution, whereas isolated fibrosis improvement remained modest. Despite being underpowered due to premature enrolment termination during the COVID-19 pandemic, the study provides important proof-of-concept that simultaneous inhibition of de novo lipogenesis and triglyceride synthesis may enhance therapeutic efficacy while mitigating metabolic adverse effects. Adverse events were mostly mild-to-moderate; unfavourable lipid profiles and suboptimal glycemic control were the most notable metabolic concerns.

### 2.10. Selonsertib ± Cilofexor ± Firsocostat —ATLAS Study

The ATLAS trial evaluated triple and dual combinations of selonsertib [apoptosis signal-regulating kinase 1 (ASK1) inhibitor], cilofexor (FXR agonist), and firsocostat (ACC inhibitor) in 392 patients with bridging fibrosis or compensated cirrhosis (F3–F4) attributable to MASH [43]. The primary endpoint—≥1-stage fibrosis improvement without worsening of NASH at 48 weeks—was not significantly met by any regimen, though cilofexor/firsocostat trended highest against placebo (21% vs. 11%; *p* = 0.17). Despite this, cilofexor/firsocostat produced a significant reduction in machine-learning-derived fibrosis score and a histological shift from F3–F4 to ≤F2 fibrosis patterns, alongside significant improvements in NAS, liver biochemistry, and noninvasive markers of inflammation and fibrosis, suggesting that a disease-modifying signal could not be fully captured by the binary histological endpoint. However, it is critical to note that selonsertib monotherapy failed to meet its primary anti-fibrotic endpoints in both the STELLAR-3 and STELLAR-4 RCTs [44]. The ATLAS study effectively marked the end of clinical development for selonsertib in MASH.

### 2.11. Semaglutide with Cilofexor/Firsocostat—WAYFIND Study

Of note, combination therapeutic strategies have recently been extended to patients with MASLD-related cirrhosis, as highlighted by the WAYFIND trial (NCT04971785), the results of which were presented at the AASLD meeting in November 2025 [45]. The combination of semaglutide (2.4mg) with cilofexor/firsocostat did not achieve the primary endpoint of fibrosis improvement with no worsening of MASH over placebo (13.7% vs 8.3%, *p* = 0.2289) using standard consensus biopsy readings in 457 patients with compensated MASH-related cirrhosis. However, when liver biopsies were reassessed using machine-learning digital pathology (PathAI analysis), the combination significantly achieved both fibrosis improvement without worsening of MASH and MASH resolution in comparison with placebo. Consistently, the active arm ameliorated the non-invasive fibrosis tests compared to the placebo arm [45].

### 2.12. Senolytic Drugs—TRUTH Study

Recently, the combination of senolytic drugs dasatinib and quercetin for 21 weeks significantly ameliorated MASH resolution and, for the first time, resulted in fibrosis improvement over placebo in MASH patients with F2-F3 fibrosis. However, the relatively small sample size (n = 31) unambiguously affects the strength of the results [46].

**Table 2:** Published randomized clinical trials of combination treatment in MASH.

RCT/Name/Mode of Action/Ref	Details of the Study	MASH Resolution Without Fibrosis Worsening	≥1-Stage Fibrosis Improvement Without MASH Worsening	Effect on Liver Fat
SYNERGY-NASH Tirzepatide/GLP-1/GIP dual receptor agonist/[30]	Phase 2b RCT, n = 190 MASH, F2–F3 fibrosis Tirzepatide vs. placebo, 52 weeks	43.6% (5 mg), 55.5% (10 mg), 62.4% (15 mg) vs. 9.8% placebo (*p* < 0.001).	55% (5 mg), 51% (10 mg), 51% (15 mg) vs. 30% placebo (*p* = NS). No significant ≥2-stage fibrosis improvement in any group.	MRI-PDFF relative reduction: −45.7% (5 mg), −41.3% (10 mg), −57.0% (15 mg) vs. placebo (all *p* < 0.001).
Survodutide/GLP-1/GCG receptor dual agonist/[31]	Phase 2 RCT, n = 293 MASH, F1–F3 fibrosis Survodutide vs. placebo, 48 weeks	47% (2.4 mg), 62% (4.8 mg) and 43% (6.0 mg), vs. 14% with placebo. ≥1-stage fibrosis improvement: 34–36% vs. 22% with placebo. Consistent efficacy in East Asian subgroup (APASL 2025).	34% (2.4 mg), 36% (4.8 mg), 34% (6.0 mg) vs. 22% placebo (*p* = NS).	≥30% relative MRI-PDFF reduction: 63% (2.4 mg), 67% (4.8 mg), 57% (6.0 mg) vs. 14% placebo (*p* < 0.001) Mean relative MRI-PDFF reduction ~67% at highest dose vs. baseline.
SYNCHRONIZE-MASLD/Survodutide/GLP-1/GCG receptor dual agonist/[32]	Phase 3 RCT, n = 216 Obese and at-risk MASLD Survodutide vs. placebo, 48 weeks	NA	NA Liver stiffness decreased by 28.7% (survodutide) and 9.2% (placebo) (*p* = 0.002), assessed by VCTE, but NS assessed by MRE.	At 48 weeks: proportion of patients with ≥30% reduction in LFC vs. baseline: 84.2% (survodutide) vs. 24.3% (placebo) (*p* < 0.0001).
IMPACT Pemvidutide/GLP-1/GCG receptor dual agonist (1:1)/[34]	Phase 2b RCT, n = 212 MASH Pemvidutide vs. placebo 24 weeks	58% (1.2 mg pemvidutide), 52% (1.8 mg pemvidutide) vs. 20% (placebo) (*p* < 0.0001)	35% (1.2 mg) and 31% (1.8 mg) vs. 26% placebo (*p* = NS) Non-invasive fibrosis markers (ELF, LSM) significantly improved vs. placebo at 24 weeks; liver stiffness decreased by −3.7 kPa (pemvidutide) and −2.2 kPa (placebo) (*p* < 0.001).	At 48 weeks, liver fat changes: −45.2% (1.2 mg), −54.7% (1.8 mg) and −8.2% (placebo) MRI-PDFF: liver fat normalization (≤5%) in 31% (1.2 mg) and 44% (1.8 mg) vs. 4% placebo (*p* < 0.001).
Efinopegdutide vs. Semaglutide/GLP-1/GCG receptor dual agonist (2:1 potency) vs. GLP-1 RA/[33]	Phase 2a active-comparator RCT, n = 145 MASLD Efinopegdutide vs. semaglutide 24 weeks	NA	NA	Relative MRI-PDFF reduction at 24 weeks: 72.7% with efinopegdutide vs. 42.3% with semaglutide (*p* < 0.001).
Retatrutide/GLP-1/GIP/GCG triple receptor agonist/[35]	Phase 2a RCT MASLD, n = 98, baseline liver fat ≥10% Retatrutide vs. placebo 24–48 weeks	NA	NA	Relative MRI-PDFF reduction at 24 weeks: −42.9% (1 mg), −57.0% (4 mg), −81.4% (8 mg), −82.4% (12 mg) vs. +0.3% placebo (all *p* < 0.001). Normal LF (<5%) achieved at 24 weeks: 27% (1 mg) (*p* < 0.05), 52% (4 mg), 79% (8 mg), 86% (12 mg) vs. 0% placebo (all except from 1mg: *p* < 0.001) Largest liver fat reduction reported for any agent in MASLD to date.
MAESTRO-NASH subgroup analysis Resmetirom + background GLP-1RA/THR-β agonist + incretin-based weight loss (background therapy) /[47]	Phase 3 subgroup analysis (13–17% of MAESTRO-NASH cohort), n = 966 MASH, F2–F3 fibrosis Resmetirom + background GLP-1RA vs. placebo 52 weeks	Resmetirom maintained full efficacy regardless of GLP-1RA co-administration; no additional benefit ≥5% weight loss associated with higher MASH resolution (50.0–56.6% vs. 27.6–33.8% without weight loss).	Similar rates in patients on vs. not on GLP-1RA background (rates ≈24–40% vs. ≈25–31% respectively).	Median MRI-PDFF relative reduction at week 52 was similar in resmetirom patients on or off GLP-1RA (~46–69% for resmetirom 100 mg). Patients with ≥5% weight loss on resmetirom 100 mg: −69% MRI-PDFF vs. −46% for <5% weight loss.
Efruxifermin + GLP-1RA (background)/FGF21 analogue + GLP-1 receptor agonist/[37]	Phase 2b RCT, n = 31 T2D + MASH, F1–F3 fibrosis Efruxifermin + GLP-1RA vs. placebo 12 weeks	NA	Liver stiffness (FibroScan-VCTE): −3.0 kPa (efruxifermin + GLP-1RA) vs. −1.1 kPa (GLP-1RA + placebo) at 12 weeks (*p* < 0.001) ELF score, Pro-C3, and FAST score significantly improved vs. placebo. Histological fibrosis: NA.	MRI-PDFF hepatic fat fraction: ~65% relative reduction with efruxifermin + GLP-1RA vs. ~10% with GLP-1RA + placebo (*p* < 0.001). Liver fat normalization (to ≤5%) achieved in a significantly higher proportion of efruxifermin-treated patients.
HEC88473/GLP-1/FGF21 single-molecule dual agonist/[38]	Phase 1b/2a RCT, n = 60 MASLD/T2DM HEC88473 vs. placebo 5 weeks	NA	NA	Relative MRI-PDFF reduction at 5 weeks: dose-dependent, up to −47.21% in the 30.6 mg cohort vs. −15.05% placebo (*p* = 0.0143). Higher proportion of >30% relative MRI-PDFF reduction in patients with baseline PDFF >8%.
COMBAT_T2_NASH Semaglutide + Empagliflozin/GLP-1RA/SGLT2i/[48]	Phase 2 RCT, n = 61 MASLD and T2DM Semaglutide + Empagliflozin vs. placebo 48 weeks	MASH resolution without fibrosis worsening: 9.5% (combination) vs. 5.6% (placebo) (*p* = NS) Significant ↓ in NAS in combination arm (−1.3) vs. placebo (−0.1) (*p* < 0.001)	NA	Significant ↓ in steatosis in combination arm (−0.38) vs. placebo (0.06), (*p* < 0.0001)
Semaglutide ± Cilofexor ± Firsocostat/GLP-1 RA + FXR agonist ± ACC inhibitor/[39]	Phase 2, open-label, proof-of-concept RCT, n = 108 MASH, F2–F3 fibrosis Semaglutide ± Cilofexor ± Firsocostat vs. placebo 24 weeks	NA	Difference in liver fibrosis by MRE between groups at week 24 (*p* = NS) Non-invasive fibrosis markers improved: FAST score reduction greater in combination arms vs. semaglutide alone; liver stiffness reduction ≥25% achieved in 50–60% (combinations) vs. 36% (semaglutide).	MRI-PDFF absolute reduction (LS mean): −9.8% to −11.0% (combination arms) vs. −8.0% (semaglutide monotherapy) (*p* < 0.05)
LEGEND Lanifibranor ± Empagliflozin/Pan-PPAR agonist ± SGLT2 inhibitor/(https://inventivapharma.com/wp-content/uploads/inventiva-documents/Inventiva-PR-Results-LEGEND-EN-03-18-2024.pdf, accessed on 7 September 2026)	Phase 2, multicentre, placebo-controlled RCT, n = 63 MASH + poorly controlled T2DM, 24 weeks Lanifibranor ± Empagliflozin vs. placebo	NA	Significant improvements in non-invasive fibrosis markers in both arms: reductions in cT1, TIMP-1, P3NP, and Pro-C3 vs. placebo (*p* < 0.05) Histological fibrosis alterations: NA.	MRI-PDFF relative reduction: −49% (lanifibranor monotherapy) and −41% (lanifibranor + empagliflozin) vs. no change with placebo (*p* < 0.05)
DUET TERN-501 ± TERN-101/THR-β agonist ± FXR agonist/[41]	Phase 2a, double-blind RCT, n = 162 Presumed non-cirrhotic MASH TERN-501 ± TERN-101 vs. placebo 12 weeks	NA	Histological fibrosis NA TERN-101 added to TERN-501 improved fibro-inflammation markers (cT1 reduction) beyond TERN-501 monotherapy.	Relative MRI-PDFF reduction at week 12: −15.4% (1 mg), −27.5% (3 mg, *p* = 0.0036), −44.8% (6 mg, vs. −4.0% placebo, *p* < 0.0001).
MIRNA Ervogastat ± Clesacostat/DGAT2 inhibitor ± ACC inhibitor/[42]	Phase 2, double-blind, double-dummy RCT, n = 255 MASH, F2–F3 fibrosis Ervogastat ± Clesacostat vs. placebo 48 weeks	Composite primary endpoint (MASH resolution without fibrosis worsening, or ≥1-stage fibrosis improvement without MASH worsening, or both) met: 66% (ervogastat 150 mg/clesacostat 5 mg) and 63% (ervogastat 300/clesacostat 10 mg) vs. 38% placebo (*p* < 0.05)	≥1-stage fibrosis improvement w/o MASH worsening: In any combination or monotherapy arm vs. placebo (*p* = NS).	MRI-PDFF: significant dose-dependent liver fat reductions across all active arms at weeks 6, 24, and 48. Proportion with ≥30% relative MRI-PDFF reduction substantially higher in combination groups vs. placebo.
WAYFIND Semaglutide + Cilofexor/Firsocostat (MASH cirrhosis)/GLP-1 RA + FXR agonist + ACC inhibitor/[45]	Phase 2, double-blind, placebo-controlled RCT, n = 457 Compensated MASH cirrhosis (F4c) Semaglutide + Cilofexor/Firsocostat vs. placebo 72 weeks	NA	13.7% (semaglutide 2.4 mg + cilofexor/firsocostat) vs. 8.3% placebo (*p* = NS), by standard pathologist reading Cilofexor/firsocostat alone vs. placebo: 20.3% vs. 8.3% (*p* = 0.0174). PathAI digital pathology: 21.0% (combination) vs. 6.0% placebo (*p* = 0.0011)—significant when AI-based reading was used. Semaglutide alone: 12.4% vs. 6.0% placebo (*p* = 0.0093) by PathAI.	NA
ATLAS Selonsertib ± Cilofexor ± Firsocostat/ASK1 inhibitor + FXR agonist + ACC inhibitor (alone or in 2-drug combinations)/[43]	Phase 2b RCT, n = 392 Bridging fibrosis/compensated cirrhosis (MASH F3–F4) Selonsertib ± Cilofexor ± Firsocostat vs. placebo 48 weeks	NA	No combination or monotherapy arm achieved significant ≥1-stage fibrosis improvement w/o MASH worsening. Cilofexor/firsocostat: numerical improvement in fibrosis scores vs. placebo; significant reductions in ELF score, liver stiffness (TE), and ALT, AST, bilirubin, bile acids, CK-18 vs. placebo.	Firsocostat monotherapy: significant MRI-PDFF reduction −3.92% (LS mean difference) vs. placebo at week 48 (*p* = 0.033).
TRUTH study Dasatinib + quercetin/Senolytic drugs/[46]	Phase 2 RCT, n = 31 MASH Dasatinib + quercetin vs. placebo 21 weeks	71% in combination arm vs. 15% in placebo (*p* = 0.011) Improvement in NAS in combination arm (−1.43) vs. placebo (−0.39), (*p* = 0.012)	47% in combination arm vs. 7% in placebo (*p* = 0.041)	NA

Abbreviations: ACC, acetyl-CoA carboxylase; AI, artificial intelligence; ALT, alanine aminotransferase; APASL, Asian Pacific Association for the Study of the Liver; ASK1, apoptosis signal-regulating kinase 1; CI, confidence interval; DGAT2, diacylglycerol O-acyltransferase 2; F4c, compensated cirrhosis stage F4; FGF21, fibroblast growth factor 21; FXR, farnesoid X receptor; GCG, glucagon; GIP, glucose-dependent insulinotropic polypeptide; GI, gastrointestinal; GLP-1, glucagon-like peptide-1; GLP-1RA, glucagon-like peptide-1 receptor agonist; HR, hazard ratio; MASLD, metabolic dysfunction-associated steatotic liver disease; MASH, metabolic dysfunction-associated steatohepatitis; MRE, magnetic resonance elastography; MRI-PDFF, magnetic resonance imaging proton density fat fraction; NA, not applicable; NS, not significant; Pan-PPAR, pan-peroxisome proliferator-activated receptor; PathAI, pathology artificial intelligence platform; RCT, randomized controlled trial; SGLT2, sodium–glucose cotransporter 2; T2D, type 2 diabetes; THR-β, thyroid hormone receptor beta; VCTE, vibration-controlled transient elastography.

## 3. Comparative Effects of Combination Regimens on Liver Fat, MASH Resolution and Fibrosis

Taken together, the RCTs summarized above reveal a heterogeneous pattern of efficacy across principal domains of histological response. Liver fat reduction shows the widest range of efficacy and the clearest separation between regimen classes. The largest effect reported for any combination schema is retatrutide, achieving 81.4–82.4% relative MRI-PDFF reduction at 8–12 mg versus a 0.3% change with placebo after 24 weeks of treatment, followed by pemvidutide (56–76%), survodutide (59–67%) and tirzepatide (41–57%), as all assessed by MRI-PDFF. Among genuine two-drug combinations, efruxifermin added to background GLP-1RA (semaglutide, dulaglutide, or liraglutide) produced the largest validated effect: 65% relative hepatic-fat-fraction reduction versus 10% with GLP-1RA-plus-placebo, with 88% of patients achieving ≥50% reduction. On the contrary, FXR/ACC combinations with or without semaglutide performed far more modestly in liver fat amelioration. Thus, across the literature, liver fat efficacy for combination regimens specifically therefore spans roughly 10% on FXR/ACC combination to 65% on FGF21 + GLP-1RA, a six-fold range, with retatrutide as the outlying leader (up to 82.4%).

As for MASH resolution, tirzepatide (SYNERGY-NASH) produced the numerically highest rates among all regimens with a paired biopsy, up to 62.4% at 15 mg (versus 9.8% with placebo) [30], with that dose representing the highest resolution rate of any single agent or combination in a biopsy-confirmed MASH trial to date. Survodutide followed closely, achieving 62% (4.8 mg), 43% (6.0 mg) and 47% (2.4 mg) resolution versus 14% with placebo [31]. Notably, the survodutide’s 4.8 mg dose matched the tirzepatide’s 15 mg for absolute MASH resolution rate. Pemvidutide (IMPACT) achieved 58% (1.2 mg) and 52% (1.8 mg) (versus 20% placebo) [34], confirming a class effect for dual agonists across the 52–62% resolution range; though this was measured at only 24 weeks, a shorter biopsy window than SYNERGY-NASH (52 weeks) or the survodutide trial (48 weeks). Collectively, GLP-1/GIP and GLP-1/GCG dual agonists therefore achieved MASH resolution in 43–62% of patients versus 10–20% with placebo, representing a 3- to 6-fold improvement over placebo, consistently significant across all trials. The DGAT2/ACC inhibitor combination (ervogastat/clesacostat, MIRNA) achieved a significant MASH resolution rate of 63% (ervogastat 150 mg + clesacostat 5 mg) and 57% (300 mg + 10 mg) (versus 9% placebo) [42]. However, although the absolute rates are numerically comparable to the best incretin doses, the clinical interpretation is partially different since the 38% placebo response was unusually high and therefore substantially narrows the true therapeutic signal. That may be at least partially attributed to COVID-19-related lifestyle changes during the trial period and the trial’s underpowering to 73% of its planned enrolment. Finally, the combination of senolytic drugs (dasatinib plus quercetin) achieved an impressive 71% MASH resolution rate (vs 15% placebo). However, the clinical impact of these results is substantially limited by the relatively small sample size (n = 31) and duration of the study (21 weeks).

Fibrosis improvement is the hardest and most clinically meaningful histological endpoint, correlated directly with liver-related outcomes, and the domain in which the current combination therapy evidence is most sobering. Among incretin-based combinations, tirzepatide produced fibrosis improvement rates of 55% (5 mg), 51% (10 mg), and 51% (15 mg) [30]—numerically the highest absolute fibrosis improvement rates across all combination trials reviewed—yet none reached statistical significance, and the effect was paradoxically non-dose-dependent despite the clearly dose-dependent MASH resolution and fat reduction. This pattern likely reflects a combination of the short 52-week biopsy window, the intrinsically slower biology of fibrosis regression relative to steatohepatitis resolution, and the relatively great improvement in the placebo arm (30%). Survodutide showed a similar pattern of 34–36% fibrosis improvement across doses versus 22% with placebo [31], a numerically meaningful 12–14 percentage-point difference that nonetheless did not reach significance. Pemvidutide (IMPACT) showed the narrowest absolute separation: 31–35% versus 26% placebo [34], which was clearly non-significant, suggesting that at 24 weeks, the anti-fibrotic signal from GLP-1/GCG agonism is too early or too modest to be detected histologically. Importantly, the other combination studies either lacked histological fibrosis assessment [35,37,41] or failed to show any benefit compared to placebo [42,43,45,49]. Of note, the most recently presented combination schema of senolytic drugs, dasatinib plus quercetin (TRUTH), resulted in significant fibrosis improvement compared to placebo, but the small size of the study (n = 31) and the short duration (21 weeks) constitute major limitations [46].

## 4. Ongoing RCTs

Since the therapeutic landscape is rapidly evolving, a few ongoing RCTs are assessing the efficacy and safety of combination regimens in MASLD/MASH (Table 3).

Notably, five of the eight ongoing RCTs employ placebo-controlled designs, two directly compare combination therapy with active monotherapy, whereas another is a single-arm study that is assessing changes from baseline without a parallel control group. An ongoing RCT (NCT05016882) is evaluating the efficacy of semaglutide and NNCF01940499, a long-acting FGF-21 analogue, in 672 MASH patients with F2-F3 fibrosis. Importantly, the primary outcome of this study is the improvement in liver fibrosis without worsening of MASH, from baseline to 52 weeks. Additionally, HM15211 (efocipegtrutide), another GLP-1/GIP/GCG triple agonist with extended half-life, is currently under evaluation in the ongoing phase 2b HM-TRIA-201 study (NCT04505436) in 215 patients with biopsy-confirmed MASH and F1-F3 fibrosis and has received FDA Fast Track designation. Another RCT (NCT05327127) will assess the effectiveness and safety of a combination therapy of pemafibrate (K-877-ER), a highly selective PPARa agonist and tofogliflozin, an SGLT-2 inhibitor, in 300 patients with MASH and liver fibrosis over a period of 48 weeks. More recently, a multicentre, prospective, placebo-controlled, double-blind, randomized, 3-arm parallel group RCT (NCT04639414) is currently evaluating, for the first time, the combination of empagliflozin plus semaglutide vs. empagliflozin or placebo in 192 patients with T2DM and MASH with F1-F3 fibrosis. The primary endpoint of the study is the histological resolution of MASH in T2DM patients without progression of fibrosis after 48 weeks of treatment. Along this line, a Japanese RCT (UMIN000045003) is assessing whether the addition of luseoglifflozin to patients with T2DM and MASH already treated with semaglutide for two weeks can ameliorate the histological activity of MASH compared to semaglutide monotherapy over a treatment period of 52 weeks. The results of the aforementioned studies are much awaited.

## 5. Limitations of Combination Therapy in MASLD/MASH

Τhe emerging clinical evidence supports the concept that rationally designed combination regimens may provide greater therapeutic benefit than single-agent approaches by targeting multiple pathogenic mechanisms simultaneously. The encouraging results reported to date have strengthened the rationale for further investigation in larger and longer-term RCTs. However, the committed efforts towards combination therapy in MASLD are accompanied by some limitations and significant challenges. First, perhaps the most fundamental unresolved question is whether the combination therapy is genuinely more effective than optimized monotherapy. Several combination regimens have failed to meet their primary endpoints in randomized controlled trials [43,50], while others were terminated before advancing further into late-stage development due to the sponsor’s decision (NCT04065841, NCT04147195 & NCT05282121). Furthermore, the vast majority of RCTs that evaluated combination therapies have been designed against placebo rather than active monotherapy. Consequently, the incremental benefit of combination therapy over optimized single-agent treatment remains uncertain. Equally important, treatment duration should be regarded as a key determinant of therapeutic response, particularly for fibrosis regression. Prolonged monotherapy may achieve histological improvements [51], whereas the conventional 48–72-week duration of most RCTs may be insufficient to fully capture these delayed anti-fibrotic effects. An additional limitation is the methodological heterogeneity among the available RCTs. Some findings originate from subgroup analyses [47] rather than dedicated combination therapy trials, others assessed add-on treatment in patients receiving background GLP-1RAs [37], and one study had a treatment duration of only five weeks [38], reducing the robustness and comparability of the available evidence.

Moreover, targeting multiple pathways carries the risk of additive side effects, drug–drug interactions, and increased treatment complexity, which may offset potential benefits. Polypharmacy carries an increased risk of adverse events and drug–drug interactions. For instance, GLP-1 RAs are associated with gastrointestinal intolerance, which may be compounded by hepatic agents that can alter bile acid signalling or lipid profiles. Although dual (GLP-1/GIP or GLP-1/GCG) and triple (GLP-1/GIP/GCG) receptor agonists appear to have an overall safety profile comparable to that of GLP-1 RAs, whether they confer improved gastrointestinal tolerability remains to be demonstrated in dedicated head-to-head comparative trials. ACC inhibitors could cause dose-dependent hypertriglyceridemia, while FXR agonists, as exemplified by obeticholic acid, carry risks of dose-dependent pruritus and adverse lipid profile changes. Resmetirom, one of only two currently approved agents for MASH, is metabolized predominantly by CYP2C8, meaning that co-administration with CYP2C8 inhibitors such as clopidogrel, repaglinide, or certain statins, medications that are rather common in MASLD patients, may raise plasma drug levels and increase adverse event risk [28,52,53]. Potential pharmacokinetic and pharmacodynamic interactions between MASLD therapies themselves also warrant careful consideration. Although clinically significant interactions among emerging MASH agents have not yet been systematically characterized, combinations involving drugs with overlapping metabolic pathways, hepatic transport mechanisms, or adverse-effect profiles may require dose optimization and close safety monitoring as combination regimens become more widely implemented. The polypharmacy burden is further compounded by the fact that most MASH patients already take multiple medications for cardiometabolic comorbidities [54]. Of importance, the early combination trials have not uncovered unexpected safety signals, but long-term data in larger, more heterogeneous populations—especially those with compensated cirrhosis—are still lacking. In addition, subcutaneous administration remains a practical limitation for semaglutide-based combination regimens in MASH, particularly given the polypharmacy already common in this population. The emergence of oral semaglutide, already approved for T2DM [55] and showing promising results in obesity and MASLD [56], offers an opportunity to overcome this barrier. More broadly, co-formulating multiple agents from an effective combination regimen into a single oral pill, as already established with fixed-dose antihypertensive/glucose-lowering combinations, could meaningfully improve adherence, which typically declines as regimen complexity increases. Recent industry consolidation reinforces this trajectory: Novo Nordisk’s 2025 acquisition of Akero Therapeutics brought the FGF21 analogue efruxifermin under the same company developing semaglutide, with leadership explicitly positioning it for combined use with GLP-1 receptor agonism. Such single-sponsor ownership of complementary mechanisms may accelerate rational combination development and eventual co-formulation in MASH. Furthermore, regulatory approval pathways for combination regimens are substantially more demanding than those applied to single-agent therapies. Health authorities generally require convincing evidence that each component possesses independent efficacy and an acceptable safety profile, while the combined regimen must additionally demonstrate clinically meaningful added benefit beyond its individual constituents. Establishing such complementary or synergistic effects considerably increases the logistical complexity, financial burden, and timelines of drug development programs. In addition, the design of combination trials is inherently challenging, often necessitating multiple active comparator arms and larger patient populations to adequately assess incremental therapeutic value. Limited accessibility and high treatment costs represent additional obstacles to the broader implementation of combination therapies, particularly in low- and middle-income countries where the prevalence of MASLD is increasing at an alarming rate [57]. To date, the economic sustainability of multidrug therapeutic approaches in MASH has not been comprehensively assessed. Moreover, the lack of robust long-term outcome data—especially regarding prevention of cirrhosis, HCC, and liver-related mortality—further complicates reliable health–economic evaluations and cost-effectiveness modelling.

## 6. Clinical Challenges of Combination Therapy in MASLD/MASH

Although combination therapy offers a compelling biological rationale, its integration into clinical care raises multiple complex and still unresolved issues. Addressing these challenges will be essential before combination approaches can be incorporated into precision-based management algorithms for MASLD. Firstly, the determination of which patients are more likely to benefit from combination treatment poses a significant clinical challenge. Combination therapy is unlikely to be necessary for the entire MASLD spectrum. Lifestyle modification remains the cornerstone of MASLD management since metabolic drivers, such as obesity, insulin resistance, T2DM, and atherogenic dyslipidemia, directly fuel disease progression. A calorie-restricted diet and regular physical exercise leading to 7–10% weight loss can reverse steatohepatitis and improve fibrosis in non-cirrhotic MASH [58,59]. Patients with early-stage disease, minimal fibrosis, or those who achieve substantial metabolic improvement through lifestyle modification may derive sufficient benefit from structured weight reduction or single-agent pharmacotherapy, particularly when administered for an adequately prolonged treatment duration [51]. In these individuals, a stepwise therapeutic approach appears most appropriate, whereby treatment is initiated with lifestyle modification and, when indicated, monotherapy, followed by reassessment of clinical and biochemical response using non-invasive markers. Combination therapy may then be reserved for patients with an inadequate therapeutic response or evidence of disease progression, mirroring the treatment paradigms established for chronic cardiometabolic disorders such as hypertension and T2DM. In contrast, individuals with advanced fibrosis, rapidly progressive liver disease, or significant cardiometabolic burden are more likely to require intensified therapeutic approaches, as monotherapy frequently fails to induce adequate histological response in these populations.

According to EASL-EASD-EASO guidelines [60], patients with a predominantly liver-centred phenotype characterized by significant fibrosis (≥F2) may benefit more from combinations incorporating liver-directed agents, such as resmetirom, whereas individuals with dominant metabolic dysfunction and T2DM may derive greater advantage from regimens centred around incretin-based therapies, predominantly GLP-1 RAs. Compelling clinical trial evidence suggests that semaglutide may be the first-line agent for obesity management in MASLD, targeting cardiovascular complications—the leading cause of death in this population [61,62]. In the STEP 1 [63] and STEP 2 [64] RCTs, once-weekly semaglutide 2.4 mg induced a body weight loss of approximately 17% in adults with obesity and nearly 11% in those with concomitant T2DM. Furthermore, the STEP UP RCT [65] demonstrated that escalating the dose to 7.2 mg weekly increases weight loss to nearly 21%, with one-third of patients losing ≥ 25% of their body weight. Beyond weight management, the landmark SELECT RCT [66] demonstrated that semaglutide reduces the risk of major adverse cardiovascular events (MACE) by 20%, all-cause mortality by 19%, cardiovascular death by 15%, and composite heart failure events by 18% in patients with overweight or obesity without T2DM. Recommended by the European Society of Cardiology for cardiovascular risk reduction, semaglutide also addresses key modifiable metabolic drivers by reducing the risk of developing T2DM by 73% and significantly improving glycemic control, lipid profiles, and blood pressure [67]. In addition to semaglutide, tirzepatide represents another highly effective first-line option for obesity management in patients with MASLD. In SURMOUNT-1 [68] and SURMOUNT-4 [69,70] RCTs, patients with obesity without T2DM achieved a substantial 21% to 22% reduction in baseline body weight at 72 weeks, which was successfully sustained with continued treatment. For patients with concomitant T2DM, the SURMOUNT-2 [71] RCT reported a robust weight loss of 13% to 16%. Beyond weight management, tirzepatide offers extensive cardiometabolic benefits by significantly improving fasting glucose, total cholesterol, triglycerides, and systolic blood pressure. However, unlike semaglutide’s established cardiovascular data, dedicated long-term data regarding its explicit impact on MACE remain pending until the completion of the event-driven SURMOUNT-MMO RCT [72]. This distinction is critical in MASLD care, where cardiovascular mortality remains the leading cause of patient death.

It is worth mentioning that, especially for MASH patients with T2DM, SGLT2 inhibitors, or flozins, represent another preferred therapy [73]. They offer well-established cardioprotective and nephroprotective benefits that directly mitigate the major extrahepatic drivers of mortality in this population [74]. Beyond systemic metabolic management, flozins reduce hepatic de novo lipogenesis and oxidative stress, showing a promising capability to halt the upstream fibrogenic cascade [75]. Consequently, they have emerged as highly compelling agents in the MASH pipeline. Ongoing clinical evaluations are exploring flozins within multi-mechanistic dual and triple combination regimens. Since a significant proportion of MASLD patients also suffer from cardiovascular diseases or chronic renal impairment, therapies with established cardiovascular and renal benefit may simultaneously improve hepatic and extrahepatic outcomes. For example, combination approaches involving GLP-1-RAs and SGLT2 inhibitors are particularly appealing because they address obesity, insulin resistance, cardiovascular risk, and hepatic steatosis concurrently.

Of importance, the increasing recognition of MASLD heterogeneity further complicates treatment allocation. Disease progression is influenced not only by obesity and insulin resistance but also by genetic predisposition, adipose tissue dysfunction [76], inflammatory signalling, and differential fibrogenic activity [77]. Variants in genes such as Patatin-like phospholipase domain-containing 3 (PNPLA3) and Transmembrane 6 superfamily 2 (TM6SF2) are associated with accelerated fibrosis progression and may eventually contribute to risk stratification models guiding therapeutic selection [78,79]. However, recent data presented at EASL 2026 indicated that targeting PNPLA3 with an antisense oligonucleotide failed to demonstrate superiority over placebo with respect to either histological fibrosis improvement or MASH resolution in homozygous carriers of the PNPLA3 rs738409 risk allele with non-cirrhotic MASH and fibrosis [80]. Despite this preliminary evidence, the concept of biological “endotyping” in MASLD is increasingly attractive. MASLD may encompass distinct biological subtypes characterized by similar baseline liver phenotypes but differing molecular signatures and patterns of disease progression. These findings support a precision-medicine approach in which biological endotyping may help identify the most appropriate combination therapy by aligning complementary pharmacological mechanisms with the dominant pathogenic pathways driving disease in each patient [81,82,83].

Furthermore, we should acknowledge that current clinical guidelines for MASLD do not recommend traditional liver-supporting supplements or medications, such as soy phospholipids, thiazolidine carboxylic acid, ornithine aspartate, or ursodeoxycholic acid (UDCA), due to a lack of robust histopathological efficacy [60]. However, since the rapid, profound weight loss induced by these GLP-1RAs or dual/triple agonists significantly elevates the risk of cholesterol supersaturation and biliary sludge, prophylactic UDCA therapy is strongly indicated during the active weight-reduction phase to prevent acute cholelithiasis and subsequent biliary complications [84]. Beyond gallbladder protection, emerging data highlight the role of the gut–liver axis in MASLD pathogenesis, where gut dysbiosis accelerates disease progression [85]. In this context, clinical evaluations suggest that UDCA may exert secondary hepatoprotective benefits by favourably modulating the gut microbiome, reinforcing the clinical value of its targeted deployment within modern MASLD combination regimens [86].

The disease stage is another critical factor for a potential combination therapy strategy, since it profoundly influences both therapeutic goals and the likelihood of response. In patients with fibrosis stages F2–F3, the principal objectives are resolution of steatohepatitis and regression of fibrosis. Current evidence suggests that combination regimens targeting complementary pathways may enhance these outcomes beyond what is achievable with single-agent therapy. Pairing systemic metabolic agents with liver-directed therapies appears especially promising in this setting, given the simultaneous contribution of metabolic dysfunction and intrahepatic injury to disease progression. In compensated cirrhosis, however, therapeutic challenges become considerably greater. As fibrosis advances, pathogenic processes increasingly involve portal hypertension [87], vascular remodelling [88], sinusoidal dysfunction [89], and persistent stellate cell activation [90,91]—mechanisms that may be less responsive to metabolic modulation alone. Crucially, no approved pharmacotherapies are currently indicated for MASLD patients with cirrhosis (stage F4). Once cirrhosis is established, management must shift to specialized hepatological care focused on systematic disease monitoring. This includes six-monthly HCC screening via abdominal ultrasound and alpha-fetoprotein (AFP), alongside endoscopic surveillance for esophageal varices [8,92]. Furthermore, splenic stiffness assessment should be used to risk-stratify patients for portal hypertension. For patients progressing to advanced decompensated cirrhosis (Child-Pugh class B/C or MELD score > 15) or liver cancer, prompt referral to a transplant centre for evaluation is required [60]. Several agents that demonstrated encouraging activity in non-cirrhotic disease, including selonsertib, emricasan, obeticholic acid, and pegbelfermin, failed to achieve primary endpoints in cirrhotic populations. Likewise, semaglutide monotherapy did not demonstrate significant histological benefit in compensated cirrhosis, although semaglutide-based combination regimens remain under active clinical evaluation (WAYFIND study). These findings suggest that advanced disease may require prolonged treatment exposure, different mechanistic targets, or entirely distinct therapeutic paradigms. Importantly, several metabolic agents—including survodutide (LIVERAGE-Cirrhosis), efruxifermin (SYMMETRY extension/Phase III program), and semaglutide-based combinations (WAYFIND)—are currently undergoing evaluation in RCTs.

More recently, the SYMMETRY RCT evaluating efruxifermin in compensated cirrhosis reported encouraging improvements in fibrosis after prolonged treatment [93], raising the possibility that treatment duration, rather than mechanistic inadequacy of individual or combination therapeutic approaches, may partly explain previous trial failures. Nevertheless, the optimal duration of combination therapy remains undefined. Most phase 2 and 3 studies have assessed histological endpoints after approximately one year of treatment, whereas the long-term durability of response and the consequences of treatment withdrawal remain largely unknown. Given that MASLD is a chronic, metabolically driven disease closely linked to obesity, insulin resistance, and systemic inflammation, discontinuation of one or more agents within a combination regimen may result in recurrent hepatic steatosis, renewed inflammatory activity, and progressive fibrosis, particularly in the absence of sustained lifestyle modification. Consequently, future studies should determine whether combination therapy is best employed as an induction strategy to achieve rapid disease control followed by maintenance with a single agent, or whether prolonged administration of selected drug combinations is required to sustain histological remission and prevent disease progression, analogous to long-term multidrug treatment strategies used for hypertension, dyslipidaemia, and T2DM.

An emerging conceptual framework could be a staged therapeutic model consisting of an induction phase followed by maintenance therapy. Under this approach, potent multidrug regimens could initially be used to achieve rapid metabolic and histological improvement, after which treatment intensity might be reduced to maintain disease remission and cardiometabolic control. Although conceptually attractive, this strategy has not yet been prospectively validated and may be used in advanced stages of the disease. Likewise, it remains unclear whether all components of a combination regimen must be continued indefinitely or whether selected agents can be safely withdrawn once therapeutic targets are achieved. On the contrary, another approach, for patients with early stages of the disease, could incorporate a single treatment strategy for a specific period and thereafter, in the absence of improvement, an additional drug could be added to the schema. A cornerstone for either approach could be non-invasive monitoring of the patients. Modalities such as vibration-controlled transient elastography, MRI-PDFF, enhanced liver fibrosis testing, and circulating biomarkers may eventually help identify patients with stable remission versus those at high risk of relapse. Of note, although statistically significant improvements in histological fibrosis were not observed, four studies reported significant reductions in liver stiffness measured by transient elastography, suggesting that non-invasive fibrosis markers may detect therapeutic benefits not consistently captured by liver biopsy [34,37,39,43]. Nevertheless, although it seems that future treatment decisions will likely rely heavily on non-invasive tools, standardized biomarker thresholds capable of reliably guiding escalation or de-escalation of therapy remain lacking. Moreover, it is important to acknowledge that histological assessment remains one of the principal limitations of MASH RCTs. Despite standardized scoring systems and central pathology review, considerable interobserver variability may influence endpoint classification and contribute to discrepancies between studies. The emergence of artificial intelligence (AI)-assisted digital pathology offers a promising strategy to improve the reproducibility, objectivity, and sensitivity of histological assessment, potentially reducing observer-dependent variability and enhancing the detection of treatment-related histological changes. To this end, as we previously outlined, in the WAYFIND study, although standard pathologist reading found no fibrosis benefit, AI-assisted (PathAI) reassessment of the same biopsies revealed significance in both fibrosis without worsening of MASH and MASH resolution compared with placebo [45]. These findings, together with the concordant improvements observed in non-invasive fibrosis markers, suggest that AI-based pathology may identify therapeutic responses that are not consistently captured by conventional histological interpretation, although further prospective validation is required.

Finally, successful implementation of multidrug therapeutic strategies will require close multidisciplinary collaboration. Hepatologists, endocrinologists, cardiologists, obesity specialists, and primary care physicians will all have essential roles in balancing hepatic efficacy against systemic metabolic benefit, cardiovascular protection, safety, and patient adherence. As the field evolves, combination therapy in MASLD will likely move toward a precision-based model in which treatment selection is individualized according to disease stage, metabolic phenotype, genetic background, overall comorbidity burden and physical performance of the patient rather than applied uniformly across all patients.

## 7. Conclusions

The emergence of effective pharmacological therapies for MASLD has fundamentally altered the therapeutic landscape and ushered in a new era in which combination therapy is increasingly viewed as a logical next step in drug development. Despite the recent approvals of resmetirom and semaglutide, a substantial proportion of patients continue to exhibit persistent steatohepatitis, residual fibrosis, or ongoing cardiometabolic risk, highlighting the limitations of single-agent approaches in a disease characterized by remarkable biological complexity. In this context, combination therapy offers a compelling opportunity to simultaneously target multiple interconnected pathways involved in metabolic dysfunction, lipotoxicity, inflammation, and fibrogenesis.

Evidence from early RCTs suggests that combinations integrating metabolic agents with liver-directed therapies can produce greater improvements in hepatic steatosis, biochemical markers, and, in some cases, histological outcomes than monotherapy alone. At the same time, significant limitations and challenges are ahead of us. Moreover, whether combination therapy will ultimately deliver true pharmacological synergy rather than additive metabolic benefits requires confirmation in adequately powered studies incorporating active comparator arms and long-term outcome assessment.

The next generation of RCTs, including studies in advanced fibrosis and compensated cirrhosis, will be critical in defining the place of combination therapy within future treatment algorithms. Ultimately, the success of combination therapy will depend not on the number of agents combined, but on the ability to match complementary mechanisms to the biological drivers of disease in the right patient at the right time. If these challenges can be successfully addressed, combination strategies may represent the most promising pathway toward achieving durable disease modification and improving long-term liver-related and cardiometabolic outcomes in patients with MASLD.

## Figures and Tables

**Table 1:** Main biological pathways targeted by emerging therapies in MASH.

Therapeutic Class	Representative Agents	Primary Target	Principal Mechanism of Action	Main Pathogenic Pathway Addressed
Metabolic -targeted therapies				
GLP-1 receptor agonists *	Semaglutide, liraglutide	GLP-1 receptor	Appetite suppression, delayed gastric emptying, weight reduction, improved insulin sensitivity	↓ Obesity, ↓ insulin resistance, ↓ lipotoxicity, ↓ systemic inflammation, ↓ hepatic fat accumulation
Dual GIP/GLP-1 receptor agonists ^#^	Tirzepatide	GIP and GLP-1 receptors	Additive to GLP-1 signalling: Potentiates GLP-1 signalling by improving adipocyte lipid-buffering capacity and peripheral insulin sensitivity independently of weight loss, ↑ insulin secretion in a glucose-dependent manner, ↓ ectopic lipid deposition	Additive to GLP-1 signalling: ↓ Adipose dysfunction, ↓ lipotoxicity and ectopic fat accumulation
Dual GLP-1/GCG receptor agonist ^#^	Survodutide, pemvidutide, efinopeglutide	GLP-1 and GCG receptor	Additive to GLP-1 signalling: ↑ hepatic fatty acid oxidation, ↑ energy expenditure, and improved insulin sensitivity increases hepatic fatty acid β-oxidation, ↓ reduces hepatic de novo lipogenesis, mobilizes hepatic triglycerides, improved mitochondrial function	Additive to GLP-1 signalling: ↓ mitochondrial dysfunction, ↑ energy expenditure
Triple GLP-1, GIP and GCG receptor agonists ^#^	Retatrutide	GLP-1/GIP/GCG pathways	GLP-1, GIP, GCG signalling	Combined pathways addressed by GLP-1, GIP, GCG as previously reported
SGLT2 inhibitors *	Empagliflozin, dapagliflozin, luseogliflozin	Renal glucose transport	Glycosuria, improved insulin sensitivity, weight reduction	↓ Hepatic fat content, ↓ glucotoxicity, ↓ metabolic stress
PPAR pan-agonists *	Lanifibranor	PPAR-α/δ/γ	Modulation of glucose and lipid metabolism, anti-inflammatory effects	↓ Steatosis, ↓ inflammation, ↓ fibrogenesis
Liver-targeted therapies				
THR-β agonists *	Resmetirom, VK2809	Thyroid hormone receptor-β	Increased hepatic fatty acid oxidation and lipid utilization	↓ Steatosis, ↓ lipotoxicity
FGF21 analogues *	Efruxifermin, pegozafermin, efimosfermin	FGFR1c/β-Klotho complex	Improved lipid metabolism, insulin sensitivity, energy expenditure	↓ Steatosis, ↓ inflammation, ↓ fibrosis
FXR agonists *	Cilofexor, tropifexor, obeticholic acid	Farnesoid X receptor	Regulation of bile acid homeostasis, lipid metabolism and inflammatory signalling	Steatosis, inflammation, fibrosis
ACC inhibitors *	Firsocostat	Acetyl-CoA carboxylase	Inhibition of de novo lipogenesis	Hepatic triglyceride accumulation, steatosis
DGAT2 inhibitors *	Ervogastat	DGAT2 enzyme	Reduced triglyceride synthesis and storage	↓ Hepatic fat accumulation
FASN inhibitors	Denifanstat, TVB-2640	FASN	Suppression of hepatic lipogenesis	↓ Lipotoxicity, ↓ steatosis
Galectin-3 inhibitors	Belapectin	Galectin-3	Modulation of macrophage activation and fibrosis	↓ Fibrogenesis, ↓ portal hypertension
sGC activators	BI 685509	NO–sGC–cGMP pathway	Improvement of sinusoidal dysfunction and portal hemodynamics	↓ Portal hypertension, ↓ vascular remodelling
Senolytics	Dasatinib + quercetin (D + Q)	Senescent hepatocytes and hepatic stellate cells (BCR-ABL/Src-family kinases via dasatinib; PI3K/AKT and BCL-2/BCL-xL survival pathways via quercetin)	Clearance of senescent cells, selective induction of apoptosis, interruption of senescence-associated secretory phenotype (SASP)–driven paracrine signalling to hepatic stellate cells	↓ cellular senescence/SASP burden, ↓ hepatic fibrogenesis

Abbreviations: ACC, acetyl-CoA carboxylase; cGMP, cyclic guanosine monophosphate; DGAT2, diacylglycerol O-acyltransferase 2; FASN, fatty acid synthase; FGF21, fibroblast growth factor 21; FGFR1c, fibroblast growth factor receptor 1c; FXR, farnesoid X receptor; GCG, glucagon; GIP, glucose-dependent insulinotropic polypeptide; GLP-1, glucagon-like peptide-1; NO, nitric oxide; PPAR, peroxisome proliferator-activated receptor; SASP, senescence-associated secretory phenotype; sGC, soluble guanylate cyclase; SGLT2, sodium–glucose cotransporter 2; THR-β, thyroid hormone receptor beta. ↑, increased/greater; ↓, reduced/lower. * These drugs have also been used in combination treatment for MASH in published RCTs. ^#^ These drugs are considered a combination therapy.

**Table 3:** Ongoing randomized clinical trials of combination treatment in MASH.

Trial/Agents	Mechanisms	Design/Population	Duration	Registration	Status/Primary Endpoint/Expected Results
Semaglutide + NNC0194-0499 (FGF21 analogue) (NCT05016882)	GLP-1 RA + long-acting FGF21 analogue	Phase 2 RCT, n = 672 MASH with F2–F4 fibrosis Semaglutide + NNC0194-0499 (FGF21 analogue vs. placebo	19 months (~76 weeks)	NCT05016882	Active, not recruiting. Primary endpoint: fibrosis improvement ≥1 stage without MASH worsening at 52 weeks. Results awaited ~2025–2026
Pemafibrate (K-877-ER) + Tofogliflozin (NCT05327127)	Selective PPARα modulator (fibrate) + SGLT2 inhibitor	Phase 2 RCT, n = 300 MASH with F1–F3 fibrosis Selective PPARα modulator (fibrate) + SGLT2 inhibitor vs. placebo	48 weeks	NCT05327127	Recruiting. Primary endpoint: histological improvement. Results pending
Semaglutide + Luseogliflozin UMIN000045003	GLP-1 RA + SGLT2 inhibitor	Phase 2 RCT (Japanese), n = ~60 T2DM patients with MASH already on semaglutide Semaglutide plus luseogliflozin vs. semaglutide	52 weeks	UMIN000045003	Active. Primary endpoint: histological MASH activity. Addition of SGLT2i to background semaglutide. Results pending
Survodutide—Phase 3 (LIVERAGE) NCT06632444/NCT06632457	GLP-1/GCG dual receptor agonist	Phase 3 RCTs: LIVERAGE (F2–F3, NCT06632444) LIVERAGE-Cirrhosis (F4c, NCT06632457) (Phase 2 completed with positive results) Survodutide vs. placebo	52 weeks	NCT06632444 NCT06632457	Not yet open/recruiting. Primary endpoint: fibrosis improvement without MASH worsening (pre-cirrhosis); liver-related events (cirrhosis). Results: not available
Efinopegdutide (MK-6024) NCT05877547	GLP-1/GCG dual receptor agonist (2:1 potency)	Phase 2b RCT, n = ~300 MASH with F2–F3 fibrosis (phase 2a vs. semaglutide completed) Efinopegdutide vs. placebo	52 weeks	NCT05877547	Active, not recruiting. Primary endpoint: histological MASH resolution and fibrosis improvement. Results pending ~2026
HM15211 (Efocipegtrutide) HM-TRIA-201 (NCT04505436)	GLP-1/GIP/GCG triple receptor agonist (long half-life)	Phase 2b RCT, biopsy-confirmed MASH, FDA Fast Track designation Efocipegtrutide vs. placebo	Approximately 48–52 weeks	NCT04505436	Active/recruiting. Primary endpoint: histological MASH resolution and fibrosis improvement. Results pending
BI 685509 + Empagliflozin (sGC activator + SGLT2i in cirrhosis) (NCT05282121)	sGC activator (NO pathway) + SGLT2 inhibitor	Phase 2 RCT, n = 80 Compensated MASH cirrhosis BI 685509 + Empagliflozin	8 weeks	NCT05282121	Percentage change in HVPG from baseline (measured in mmHg) after 8 Weeks of treatment
Combat_T2_NASH_002 Empagliflozin + semaglutide vs. empagliflozin vs. placebo	GLP-1 RA + SGLT2 inhibitor vs. SGLT2 inhibitor	Phase 4 RCT, n = 192 MASH patients with T2DM Semaglutide + empagliflozin vs. empagliflozin vs. placebo	48 weeks	NCT04639414	Histological resolution of MASH without worsening of fibrosis

Abbreviations: FGF21, fibroblast growth factor 21; GCG, glucagon; GIP, glucose-dependent insulinotropic polypeptide; GLP-1, glucagon-like peptide-1; HVPG, hepatic venous pressure gradient; MASH, metabolic dysfunction-associated steatohepatitis; NO, nitric oxide; PPAR, peroxisome proliferator-activated receptor; RA, receptor agonist; RCT, randomized controlled trial; sGC, soluble guanylate cyclase; SGLT2, sodium–glucose cotransporter 2; T2DM, type 2 diabetes mellitus.

## Data Availability

No new data were created or analyzed in this study. Data sharing is not applicable to this article.
